# The impact of the COVID-19 pandemic on nursing home professionals: results of the RESICOVID project

**DOI:** 10.1186/s41155-023-00284-w

**Published:** 2024-03-19

**Authors:** Carlos Dosil-Díaz, Sacramento Pinazo-Hernandis, Arturo X. Pereiro, David Facal

**Affiliations:** 1 Department of Developmental and Educational Psychology, University of Santiago de Compostela, Santiago de Compostela, Spain; 2 Department of Social Psychology, University of Valencia, Valencia, Spain

**Keywords:** Burnout, COVID-19, Nursing homes, Formal caregivers, Psychological impact

## Abstract

**Objectives:**

The situation caused by the COVID-19 pandemic has had an impact on the mental, physical, and social health of nursing home staff. The operations and protocols of long-term care facilities had to be adapted to a new, unforeseen, and unknown situation in which a devastating and highly contagious disease was causing large numbers of deaths. The aim of this study was to determine the cumulative impact of the COVID-19 pandemic on care, technical, coordinating-supervisory, and managerial staff working in nursing homes.

**Methods:**

Correlation analysis and between-group comparisons were carried out to study the relationship between burnout scores, emotional balance, and organic and behavioral symptoms.

**Results:**

The results indicate high levels of burnout and psychological exhaustion. Management professionals displayed higher levels of organic and behavioral symptoms than other professional categories in the same care settings. Despite this negative symptomatology, most professionals showed a positive emotional balance.

**Conclusion:**

The need to develop intervention programs to improve the mental, physical, and occupational health of the staff in nursing homes, considering the needs of different professional categories, is emphasized.

## Introduction

Coronavirus disease (COVID-19) emerged in the city of Wuhan in China in 2019, spreading globally and becoming an international public health problem. In the context of the global crisis, healthcare workers were the first line of defense to combat the disease, but they face a health emergency with poor equipment and working conditions, which has an impact on their mental health (Boluarte et al., 2020). In Spanish nursing homes for older adults, the number of deaths exceeded 31,000 residents (Mateos et al., 2020), representing the population most severely affected by the pandemic. Professionals working in long-term care facilities such as nursing homes were forced to make important changes in their organization to mitigate or prevent these problems (Pereiro et al., 2021; Pinazo-Hernandis et al., 2022).

Burnout is common in professionals who work in direct contact with people such as care professionals, usually working in environments with high demand, low resources, low levels of recognition, and high levels of emotional involvement (Navarro et al., 2022; Organisation for Economic Co-operation and Development, 2020). It is defined as an inadequate response to chronic job stress characterized by symptoms such as depersonalization, high levels of emotional exhaustion, and low levels of personal fulfillment at work (Gil-Monte, 2003; Woodhead et al., 2016); and it is a mediating variable between perceived stress and its consequences (Gil-Monte, 2003). The relationship between the working conditions of health and care professionals, their coping with prolonged stress situations and their mental health status is well known. Extended stress influences general health and, specifically, the appearance of depressive symptoms and other mental disorders such as anxiety and sleep disturbance. It also reduces personal well-being and hinders the optimal functioning of the organism (Ludick & Figley, 2017). In a study involving long-term care nursing professionals, VonDras et al. (2009) identified a large number of specific workplace stressors, including stress due to coworkers, resulting from intrapersonal processes, interaction with residents, management-related factors, and due to demands from the resident’s families.

The COVID-19 pandemic has not only affected the health of people who have had the disease, whether in hospitals, homes, or long-term care facilities; it has also affected professional caregivers. Studies conducted during the COVID-19 pandemic indicate that during this health crisis, professional caregivers have developed symptoms of depression, stress, anxiety, and fatigue (Alcalá, 2020; Greenberg et al., 2020; Lu et al., 2020; Yang et al., 2020) and are at risk of developing post-traumatic stress disorder (Blanco-Donoso et al., 2020; Canal-Rivero et al., 2022). A meta-analysis conducted in 2020 revealed a high prevalence of depressive and anxiety symptoms in health and social care professionals, especially in female nurses (Pan et al., 2020; Pappa et al., 2020). Furthermore, 51.7% of front-line professionals were infected with the COVID-19 virus and had physical symptoms such as fever, cough, fatigue, and headache (15.1%) (Gholami et al., 2021).

The sustained stress experienced during the COVID-19 pandemic is likely to cause more people to question their work in care settings. Recommendations for the reduction of stress and psychological distress in health professionals during the pandemic included attention to basic needs (sleep, food, rest), a clear distribution of tasks, good team communication, and the use of psychological support (Petzold et al., 2020). Organisation for Economic Co-operation and Development (2020) stresses the importance of improving working conditions in the long-term care field, promoting better use of skills, and investing in prevention to increase retention rates in the long-term care workforce.

Although it is well known that the most difficult moments of the COVID-19 pandemic occurred in the first and second waves (Pereiro et al., 2021), when nursing home professionals were exposed to high levels of stress, the present study aimed to determine the cumulative levels of distress during the fifth wave of the pandemic, considering that in all nursing homes, the impact of COVID-19 was not equal, nor were the resources they had to cope with it, and some of them experienced several episodes of contagion over time. The objective was to determine the intermediate-term impact of the COVID-19 pandemic on the mental and physical health of nursing home professionals by analyzing differences in effects in different professional categories.

## Methods

### Participants

A total of 580 professionals completed the questionnaire, 77 of whom were excluded due to errors in the recording of data or omissions in the answers. The final sample consisted of 503 workers (85% women of mean age 38.5 years, S.D. = 10.35).

Of the sample, 70.3% of the participants were from the Valencian Community and 29.7% from Galicia, and different healthcare professional profiles were represented (care staff and auxiliary nursing staff, 275 participants, 54.7% of the sample; professional technical staff, including nurses, medicine doctors, psychologists, occupational therapists, social workers, and social educators, 148 participants, 29.4%; coordinators-nursing supervisors, 36 participants, 7.2%; and directors, 44 participants, 8.7%) (see Table 1 for further details).

**Table 1:** Sociodemographic data and characteristics of the participants

Variable	Auxiliary	Technical staff	Coordinator- supervisor	Director	Total
Age group					
18–26	40 (14.5%)	19 (12.8%)	5 (13.9%)	6 (13.6%)	70 (13.9%)
27–34	70 (25.5%)	31 (20.9%)	9 (25.0%)	10 (22.7%)	120 (23.9%)
35–42	71 (25.8%)	31 (20.9%)	11 (30.6%)	10 (22.7%)	123 (24.5%)
43–50	62 (22.5%)	47 (31.8%)	5 (13.9%)	7 (15.9%)	121 (24.1%)
51–66	32 (11.6%)	20 (13.5%)	6 (16.7%)	11 (25.0%)	69 (13.7%)
Gender					
Female	248 (90.2%)	118 (79.7%)	26 (72.2%)	36 (81.8%)	428 (85.1%)
Male	27 (9.8%)	30 (20.3%)	10 (27.8%)	8 (18.2%)	75 (14.9%)
Autonomus community					
Galicia	81 (29,5%)	40 (27,0%)	9 (25,0%)	20 (45,5%)	150 (29.8%)
Valencia	194 (70,5%)	108 (73,0%)	27 (75,0%)	24 (54,5%)	353 (70.2%)
Educational level					
University education	11 (4.0%)	109 (73.6%)	20 (55.6%)	42 (95.5%)	182 (36.2%)
Secondary education	221 (80.4%)	32 (21.6%)	12 (33.3%)	2 (4.5%)	267 (53.1%)
Basic education	43 (15.6%)	7 (4.7%)	4 (11.1%)	0 (0%)	54 (10.7%)
Years worked in nursing home					
< 1 year	54 (19.6%)	30 (20.3%)	8 (22.2%)	11 (25.0%)	103 (20.5%)
1–3 years	66 (24.0%)	35 (23.6%)	10 (27.8%)	6 (13.6%)	117 (23.3%)
4–6 years	59 (21.5%)	27 (18.2%)	8 (22.2%)	5 (11.4%)	99 (19.7%)
7–9 years	19 (6.9%)	21 (14.2%)	3 (8.3%)	9 (20.5%)	52 (10.3%)
> 10 years	77 (28.0%)	35 (23.6%)	7 (19.4%)	13 (29.5%)	132 (26.2%)
COVID-19 infection in older adults					
Yes	192 (69,8%)	115 (77.7%)	27 (75.0%)	34 (77.3%)	368 (73.2%)
No	83 (30.2%)	33 (22.3%)	9 (25.0%)	10 (22.7%)	135 (26.8%)
Professional casualties					
Yes	258 (93.8%)	143 (96,6%)	33 (91.7%)	41 (93.2%)	475 (94.4%)
No	17 (6.2%)	5 (3.4%)	3 (8.3%)	3 (6.8%)	28 (5.6%)

### Materials

The assessment instrument gathered sociodemographic and job-related data on variables such as age, sex, region (Galicia/Valencian Community), number of years employed in the nursing home, job position, and educational level. We recorded whether the nursing home had had cases affected by COVID-19 and whether there had been professional leave due to infection or close contact with infected people. The assessment also included the Questionnaire for the Evaluation of Occupational Burnout Syndrome at Work (“Cuestionario para la Evaluación del Síndrome de Quemarse por el Trabajo”, CESQT in the original version in Spanish) (Gil-Monte, 2011), the Positive and negative affectivity questionnaire (PANAS) (Watson et al., 1988) and the Organizational Behavior Psychosocial Research Unit (“Unidad de Investigación Psicosocial de la Conducta Organizacional”) (UNIPSICO) battery (Gil-Monte, 2016).

The CESQT evaluates burnout syndrome, understood as a response to chronic work stress that originates in professionals dedicated to working with people. It was designed in terms of responses to work stress among workers in different professions (educational professionals, healthcare professionals, etc.) (Gil-Monte, 2011). It is composed of 20 items distributed in four subscales: job satisfaction (desire to achieve work goals because they are a source of personal satisfaction), psychological exhaustion (presence of emotional exhaustion and physical due to problems at work), indolence (presence of negative attitudes of indifference and cynicism towards the users of the organization) and guilt (feelings of guilt that the person has by negative behavior and attitudes developed at work, especially towards the people with whom labor relations are established). It has a 5-point response format. The scores obtained can be interpreted according to a percentile table indicating different levels of occupational burnout (critical, high, medium, low, and very low). The test was developed in a Spanish context and validated with large samples from Spain, Argentina, Brazil, Chile, Portugal, Mexico, Colombia, Costa Rica, Peru, and Uruguay.

The PANAS questionnaire assesses the relationship between positive and negative dimensions of affect. It is composed of 20 items grouped into 2 subscales describing 10 positive and 10 negative emotions (Watson et al., 1988). Each item is answered using a Likert-type scale with 5 response options (not at all, very little, somewhat, quite a lot, very much). Each subscale has scores ranging between 10 and 50, with high scores on each of the subscales suggesting high positive emotional balance. This scale has been shown to be significantly correlated with depression, anxiety, and well-being, among others, and its Spanish version has been successfully used to measure emotional reactions to the COVID-19 pandemic (Facal et al., 2021).

The UNIPSICO battery was designed to assess psychosocial factors and risks at work and their consequences. It collects information on organic symptoms in items 1, 2, 3, 4, 5, 6, 7, 8, and 9 and behavioral symptoms in items 10, 11, 12, and 13. Within the organic symptoms, information on respiratory problems, palpitations, chest pains, stomach pains, anxiety, dizziness and vertigo, headaches, sleeping problems, and muscle pains is collected. Within behavioral symptoms, anxiety-related behavioral responses such as increased use of tobacco, alcohol, and drugs and seeking professional support are assessed. It uses a 5-point scale ranging from 0 (never) to 4 (very frequently: every day). The instrument was developed and validated between 2000 and 2005 in the Psychosocial Research Unit of Organizational Behaviour, University of Valencia (Gil-Monte, 2016).

### Procedure

The study presented here is part of the RESICOVID research project, conducted in the Psychology Faculties at the Universities of Santiago de Compostela and Valencia. The aim of this project was to study the impact that COVID-19 was having on both older adults and professionals working in long-term care centers. Previous research within this project found that the isolation measures imposed had a strong impact on the physical, cognitive, and emotional health of older adults (Pereiro et al., 2021; Pinazo-Hernandis et al., 2022).

In this study, we collected data from professionals working in nursing homes during the fifth wave of the COVID-19 pandemic in Spain (June–September 2021). Several nursing homes in Galicia and Valencian Community (Spain) were contacted to request the collaboration of the professionals in the study. They were provided a link to a questionnaire to be completed anonymously (using Microsoft Office Forms). In Galicia, the questionnaire was distributed through the associates of the Galician Association of the Dependency Sector (AGASEDe), while one of the largest groups of participants was in Valencian Community.

The nursing homes included in the study have similar characteristics and are mainly centers with a large capacity (between 100 and 150 residents). Professionals who were active during the entire pandemic were invited to participate in the study by the care home directors under the supervision of the research team. The questionnaires were distributed electronically, using Microsoft Office Forms from the University’s institutional account.

The study was conducted in accordance with the principles of the Declaration of Helsinki. Informed consent was obtained from the respondents at the beginning of the questionnaire. The data in the questionnaire were treated anonymously. Only the research teams from the University of Santiago de Compostela and the University of Valencia had access to the contents of the questionnaires. The project was approved by the Ethics Committee of the University of Santiago de Compostela (reference USC-11/2020).

### Data analysis

A descriptive analysis of the variables under study was performed. Qualitative variables were described by absolute and relative frequencies. Quantitative variables were described by means and standard deviations. Pearson's bivariate correlation analysis was performed to study the relationship between the results of burnout, emotional balance, and organic and behavioral symptoms. Between-group comparisons were made according to professional category (care staff, technical staff, coordinators-nursing supervisors, directors) using one-way ANOVA with Bonferroni post-hoc tests. All analyses were performed using the SPSS-21 statistical program, and a significance level of *p* < 0.05 was applied in all tests.

## Results

The results of the CESQT test are shown in Tables 2 and 3. The highest proportions of the total CESQT results were located in the high and medium levels of burnout, with a mean of 1.34 and a standard deviation of 0.11. In the Job satisfaction subscale, the proportions are mainly located in the medium, low, and very low levels, with a mean value (and standard deviation) of 2.8 (0.71). In the psychological exhaustion subscale, the highest percentages are mainly at the high and medium levels, with a mean value (and standard deviation) of 2.06 (0.87). In the indolence subscale, the highest proportion is at the medium level with a mean value (and standard deviation) of 0.80 (0.57). Finally, in the guilt subscale, the highest proportion was at the low level.

**Table 2:** Mean, standard deviation (in parentheses), 95% confidence interval, and correlations for study variables

	M (SD) CI	1	2	3	4	5	6	7	8
1. CESQT Total	1.34 (0.11) 1.33–1.35	–							
2. CESQT Job satisfaction	2.80 (0.71) 2.74–2.86	− 0.76**	–						
3. CESQT Psychological exhaustion	2.06 (0.87) 1.9–2.14	0.84**	− 0.45**	-					
4. CESQT Indolence	0.80 (0.57) 0.75–0.85	0.70**	− 0.37**	0.41**	−				
5. CESQT Guilt	0.74 (0.62) 0.69–0.80	0.41**	− 0.25**	0.33**	0.45**	–			
6. PANAS Emotional balance	1.84 (0.37) 1.81–1.87	− 0.07	0.05	− 0.06	− 0.08	− 0.04	–		
7. UNIPSICO Organic symptoms	12.6 (6.66) 12.01–13.18	0.03	− 0.03	0.01	0.03	0.03	− 0.39**	–	
8. UNIPSICO Behavioural symptoms	2.84 (2.4) 2.62–3.06	0.04	− 0.06	− 0.01	0.06	0.02	− 0.36**	0.58**	–

**p < .01

**Table 3:** Frequencies and percentages of the different score ranges in the CESQT test

	Job satisfaction		Psychological exhaustion		Indolence		Guilt	
Critical	0	0%	33	6,6%	32	6,4%	37	7.4%
High	54	10.74%	265	52.7%	78	15.5%	92	18.3%
Medium	188	37.4%	168	33.4%	253	50.3%	167	33.2%
Low	204	40.6%	25	5%	65	12.9%	204	40.6%
Very low	57	11.3%	11	2.2%	75	14.9%	0	0%

The results of the PANAS and UNIPSICO questionnaires are also shown in Table 2. The sum of the positive PANAS items yielded a mean score of 36.5 (s.d. = 6.95), while the sum of the negative PANAS items yielded a mean score of 23.03 (s.d. = 7.8). The percentage of participants with a positive emotional balance according to the PANAS data is 81.7% and the percentage of a negative or neutral emotional balance is 18.3%. The UNIPSICO results for organic symptoms produced a mean score of 12.6 (s.d. = 6.6). For behavioral symptomatology, the mean score was 2.8 (s.d. = 2.4).

### Group differences between professional categories in the evaluation of burnout, emotional balance, and organic and behavioral symptoms

Regarding the between-groups differences according to a professional category, there were significant differences between groups in the total measure of burnout, in the indolence subscale, and both in organic and behavioral symptoms (see Table 4). The post-hoc comparison showed significantly higher levels of Organic symptoms in directors than in healthcare staff and technical professionals, and higher levels of behavioral symptoms than in technical professionals.

**Table 4:** Mean, standard deviation (in parentheses), 95% confidence interval, and one-way analyses of variance in the different professional categories

	1. Auxiliary	2. Technician	3. Coordinator/supervisor	4. Director	*F*(3,499)	Post-hoc
CESQT Total	1.34 (0.11) 1.33–1.35	1.31 (0,11) 1.30–1.34	1.36 (0.13) 1.32–1.40	1.36 (0.13) 1.32–1.40	2.84*	
CESQT Job satisfaction	2.80 (0.68) 2.73–2.89	2.86 (0.72) 2.74–2.98	2.73 (0.77) 2.47–2.99	2.60 (0.84) 2.35–2.86	1.59	
CESQT Psychological exhaustion	2.07 (0.86) 1.96–2.17	1.96 (0.86) 1.82–2.10	2.26 (0.93) 1.94–2.57	2.19 (0.94) 1.90-2.47	1.51	
CESQT Indolence	0.84 (0.59) 0.77–0.91	0.69 (0.52) 0.61–0.78	0.86 (0.60) 0.65–1.06	0.90 (0.59) 0.75–1.08	2.79*	
CESQT Guilty	0.77 (0.65) 0.69–0.84	0.70 (0.61) 0.60–0.80	0.82 (0.63) 0.60–1.03	0.72 (0.60) 0.53–0.90	0.59	
PANAS Emotional balance	1.86 (0.35) 1.82–1.90	1.81 (0.39) 1.74–1.87	1.92 (0.28) 1.82–20.01	1.72 (0.45) 1.59–1.86	2.55	
UNIPSICO Organic symptoms	12.11 (6.41) 11.35–12.87	12.45 (6.67) 11.7–13.54	13.42 (6.97) 11.06–15.77	15.5 (7.42) 13.24–17.76	3.53*	1,2 < 4
UNIPSICO Behavioral symptoms	3.03 (2.58) 2.72–3.33	2.34 (1.98) 2.02–2.67	3.06 (2.95) 2.06–4.05	3.18 (2.68) 2.36–4.00	2.91*	2 < 4

*p < .05

## Discussion

The aim of this study was to determine the impact of COVID-19 on the self-perceived mental, physical, and social health of professionals caring for older adults living in nursing homes. The results indicate high levels of burnout and organic symptomatology, together with positive emotional balance. The review of the literature establishes high levels of stress and impact on the health of workers in long-term care centers during the pandemic. Our study supports these patterns of results, emphasizing the implications for professionals in management positions.

Directors displayed higher levels of organic and behavioral symptomatology. Many of the research studies that have emerged in relation to the pandemic have focused on the physical and emotional burden suffered by care professionals, such as nursing assistants, health professionals, and even non-professional caregivers (El Haj et al., 2020; Torrente et al., 2021), but this research highlights the psychological burden suffered by professionals in management positions, who have largely been responsible for acting as intermediaries between the decisions of the health authorities and the practical needs of each center (Marshall et al., 2021; Scopetti et al., 2021). The interviews conducted by Pinazo-Hernandis et al. (2022) showed the great complexity of the management of human resources in centers during the pandemic. Perceived responsibility is a variable that is correlated with the feeling of being overloaded with work (Svergun & Fairlie, 2020).

One striking finding is the higher proportion of professionals with a positive emotional balance. These data indicate that when positive affect is evaluated, numerous professionals manifest emotions such as enthusiasm, activity, or attention among other factors of positive affectivity, while negative affect is present to a lesser extent. In this respect, Martínez et al. (2014) demonstrated that an adverse situation can lead to greater commitment and dedication to fighting adversity, trying to give meaning to life, and setting long-term life goals. In the context of the COVID-19 pandemic, Canal-Rivero et al. (2022) did not find significant longitudinal increases in stress responses among health-care workers. These data may seem contradictory to the results obtained in burnout, with a high percentage of healthcare and nursing home professionals presenting high and medium levels of burnout and psychological exhaustion (Navarro-Prados et al., 2023; Torrente et al., 2021). One possible explanation is that both measure different facets as the burnout questionnaire focuses specifically on the work environment while the emotional balance questionnaire collects information from different areas of the person’s life, with different emotions present with different emotional valence.

The COVID-19 pandemic has stressed the need for mental health prevention and psychological care interventions for nursing home workers that can promote their biopsychosocial health and reduce or eliminate their burnout levels (Navarro et al., 2022). The aim is to improve the perception, interpretation, and re-evaluation of stressful situations in the work context, reducing professional burnout, preventing future health complications, and increasing the personal resources of workers (Gil-Monte, 2003; Organisation for Economic Co-operation and Development, 2020; Petzold et al., 2020). The results of our study add evidence to this need.

## Conclusions

The different professional categories of staff in the long-term care centers presented high or medium symptoms of burnout. Management staff displayed a higher incidence of negative organic and behavioral symptoms. The sustained stress suffered by professionals in nursing homes during the COVID-19 pandemic until its fifth wave (March 2020 to September 2021) has increased the physical and emotional burden of the professionals. Despite this negative symptomatology, most of the professionals showed a positive emotional balance.

The results obtained show stress was maintained over time. To the best of our knowledge, this is the first study with an intermediate-term perspective in a sample of professionals. The results highlight the need to implement programs to prevent future problems in the mental and organic health of professionals in nursing homes and to intervene when the situation has already occurred with psychological care programs. The programs should target the various professional categories in different ways according to their needs.

## Data Availability

The datasets generated during and/or analyzed during the current study are available from the corresponding author upon reasonable request.

## Abbreviations

COVID-19: COronaVIrus Disease of 2019
CESQT: Questionnaire for the Evaluation of Occupational Burnout Syndrome at Work.(“Cuestionario para la Evaluación del Síndrome de Quemarse por el Trabajo” in Spanish)
AGASEDe: Galician Association of the Dependency Sector (“Asociación Galega no Sector da Dependencia” in Galician)
PANAS: Positive and Negative Affectivity Questionnaire
UNIPSICO: Organizational Behavior Psychosocial Research Unit (“Unidad de Investigación Psicosocial de la Conducta Organizacional” in Spanish)
